# Gigapixel confocal imaging using a massively parallel optical probe array with single directional infinite scanning

**DOI:** 10.1038/s41598-020-64602-3

**Published:** 2020-05-06

**Authors:** Ryung Shin, Woojae Choi, Taekyung Kim, Donghyun Kim, Runjia Han, Kyungjin Lee, Nahyun Won, Shinill Kang

**Affiliations:** 1 School of Mechanical Engineering, Yonsei University, Seoul, 03722 South Korea; 2 National Center for Optically-assisted Mechanical Systems, Yonsei University, Seoul, 03722 South Korea; 3 Departments of Systems Engineering, University of Texas at Dallas, Richardson, TX 75080 USA

**Keywords:** Imaging and sensing, Micro-optics

## Abstract

Here we demonstrate high-throughput gigapixel confocal imaging using a massively parallel optical probe array with single directional infinite scanning. For implementation of the single directional infinite scan with high lateral resolution, a parallelogram array micro-objective lens module, composed of two wafer-level microlens arrays, is proposed to generate a massively parallel optical probe array for integration into the confocal imaging system, including an objective-side telecentric relay lens with a low-magnification. To test the feasibility of the proposed system with single directional infinite scanning, we designed and constructed a confocal imaging system using a parallelogram array of multi-optical probes with a massively parallel array size of 200 × 140. The constructed system provides a full width-half maximum lateral resolution of 1.55 μm, as measured by the knife-edge detection method, and a field-of-view width of 28.0 mm with a sampling interval of 1 μm/pixel.

## Introduction

Nano/micro-fabrication technologies for applications such as displays, semiconductors, biosensors, and energy devices have progressed, such that they are now able to produce ultra-fine feature sizes over a large-structured area with high integration^1–10^. As applications in nano/micro fabrication have advanced, there has been increasing demand for inspection technologies to enable cost-effective, large-area, high-resolution imaging^11–14^. It is difficult to meet the resolution and imaging area requirements simultaneously during optical inspection under high magnification. An optical system satisfying both the high-resolution and large-area conditions must include a wide imaging circle and high numerical aperture (NA) optical components; thus, it is vulnerable to aberrations. Moreover, such a design is highly complicated, with a high implementation cost.

Confocal imaging has been suggested as a solution to overcome resolution limitations^15–17^. However, there are also restrictions in terms of the scalability of the imaging area for confocal imaging systems employing beam scanning schemes, such as galvanometer mirrors^18–20^, polygon mirrors^21,22^, and Nipkow disks^23,24^, due to the narrow field-of-view (FOV), as determined by the field number of the high magnification objective lens.

To achieve a large FOV with high lateral resolution, we proposed a multi-optical probe confocal imaging system in our previous study^25^. The system overcomes the limitation of the scalability caused by the field number of the objective lens using multi-optical probes generated by the microlens array. However, the use of microlens array cannot apply to practical use because of its short working distance. The system aims to increase the working distance of an objective microelens array by exploiting the confocal effect to considerably reduce loss of lateral resolution. The confocal effect of the proposed system is created by optical conjugation between a micro-objective lens module (μOLM) and the aperture of the relay lens, which is used as a pinhole. This optical system has the advantages of reduced unit cost for optical system integration and simplified scaling. However, the proposed system using the step-and-repeat scanning method requires an expensive high-performance stage with a long travel range and precise movement resolution. In addition, scanning along the raster line has presented some drawbacks with respect to increased scanning throughput.

In this study, we demonstrate a high-throughput, gigapixel confocal imaging system using massively parallel multi-optical probes with single directional infinite scanning. For application to a single directional scan, a parallelogram array multi-optical probe (Pa-MOP) is proposed. An imaging system using a Pa-MOP can acquire optical information corresponding to the width of the transverse direction of a lens array with single directional infinite scanning. A parallelogram array micro-objective lens module (Pa-μOLM) composed of two microlens arrays based on a PA-MOP was designed for integration into a multi-optical probe confocal imaging system. The Pa-μOLM can be adjusted to the sampling interval in the transverse or scanning direction, by arranging the microlens and synchronizing the sample moving speed with the image acquisition frequency of the camera, making it suitable for application to large-area, high-throughput inspection systems.

The proposed system can achieve a higher resolution using a smaller aperture, without considering the optical performance of the relay lens, through generation of the confocal effect by optical conjugation of the Pa-μOLM and the aperture of an objective-side telecentric relay lens. This configuration allows to apply a low-magnification relay lens to the system, to simultaneously ensure high-lateral resolution and guarantee large-area scalability. Furthermore, the use of a low-magnification relay lens has the advantages of increasing the density of optical probes by increasing the sampling size of the image sensor and being secured adequate image brightness despite the decreased aperture diameter.

To test the feasibility of high-throughput gigapixel imaging using the proposed system with a single directional scan strategy, we designed and constructed a multi-optical probe confocal imaging system integrated with a PA-μOLM with a massively parallel array size of 200 × 140. The constructed system provided a FOV width of 28.0 mm, sampling interval of 1-μm/pixel, and WD of 445.0 μm. The Pa-μOLM was composed of two wafer-level microlens arrays with a parallelogram arrangement, i.e., an intermediate microlens array (IMA) and an objective microlens array (OMA), integrated on either side of a glass substrate. An ultraviolet (UV)-imprinting process was applied to fabricate the wafer-level Pa-μOLM, to secure high durability, mass producibility, and high geometrical accuracy^26^. The module was integrated into an objective-side telecentric relay lens with a low-magnification to create a confocal imaging system with single directional infinite scanning. The full width-half maximum (FWHM) of the lateral resolution measured by the knife-edge detection method was 1.55 μm. Imaging tests of the USAF 1951 resolution target and the chrome-deposited mask were performed to evaluate the optical performance of the system. Finally, gigapixel confocal imaging was conducted using an actual large-area thin-film transistor (TFT)-bottom glass for a liquid crystal display (LCD), to verify the practical utility of the proposed system for inspecting actual large-area electronic circuits with high throughput.

## Results

### Confocal imaging strategy using a parallelogram array of multi-optical probes with single directional infinite scanning

**Figure 1:**
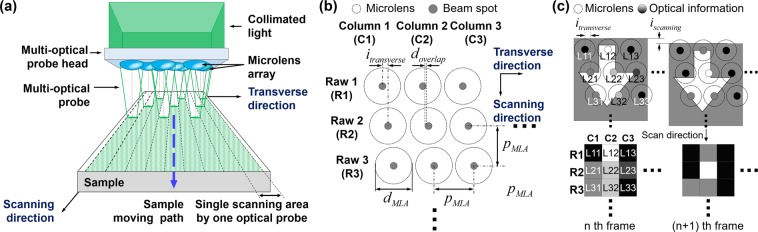
Single directional infinite scanning for high-throughput gigapixel confocal imaging using a parallelogram array multi-optical probe (PA-MOP). (**a**) Conceptual diagram of the scan strategy for multi-optical probe imaging, (**b**) Pa-MOP schematic diagram for applying a single directional scan based on a multi-optical probe, and (**c**) optical information acquisition result per probe for a single directional scan and schematic diagram of the information change per frame.

The imaging system in the previous study was limited in terms of its imaging throughput due to use of the step-and-repeat scan method, which followed a raster line with a high-performance stage. To overcome this limitation, a single directional infinite scanning method is proposed, as shown in Fig. 1(a), which has a large-area imaging system using a multi-optical probe generated by a microlens array. An imaging system using a multi-optical probe can acquire optical information corresponding to the width of the transverse direction of the lens array with single directional infinite scanning. A sequential frame can be created through the acquisition of optical information along the scanning direction and merged to obtain a gigapixel image with high throughput.

Our proposed scanning method with a massively parallel optical probes in a proposed confocal imaging system has the following advantages. First, design of the microlens array is simple because the required imaging area and sampling interval of the final image are determined intuitively by the number of microlenses and the spaces between them. Second, the confocal optics configuration prevents cross-talk between microlenses, enabling the arrangement of multi-optical probes with a high density. Third, as the alignment between the optical probe and the pinhole for the confocal configuration is unnecessary, no restriction is imposed on microelens arrangement and shape.

To allow the application of a single directional scan strategy to a confocal imaging system, a Pa-MOP is proposed, as shown in Fig. 1(b). The Pa-MOP is composed of a lens array in the transverse direction with a predetermined pitch (*p*_*MLA*_) and diameter (*d*_*MLA*_), arranged perpendicular with respect to the scanning direction, and a lens array in the scanning direction spaced according to a sampling interval (*i*_*transverse*_) appropriate for each row.

The number of lenses in a column is set to be larger than the value obtained by dividing the *p*_*MLA*_ by *i*_*transverse*_, such that a non-scanned area does not form between the lenses in the transverse direction during scanning. In addition, *i*_*transverse*_ is determined by considering the overlap of the beam spots (*d*_*overlap*_) constituting the column. The sampling interval in the scanning direction (*i*_*scanning*_) can be adjusted according to the line scan frequency (μm/pixel).

Figure 1(c) shows a schematic diagram of the method for acquiring optical movement information for each frame of a single directional scan using PA-MOP. Because the optical information acquired from the probe is the brightness value of a pixel constituting the reconstructed image, the optical information simultaneously acquired using the Pa-MOP is added to one frame for easy image recombination. Consecutively numbered frames are created using updated optical information with movement along the scanning direction, and a reconstructed image is obtained using a mapping algorithm by arranging all pixels in the frames in their pre-designated positions.

### Design of a gigapixel confocal imaging system using a parallelogram array micro-objective lens module with single directional infinite scanning

**Figure 2:**
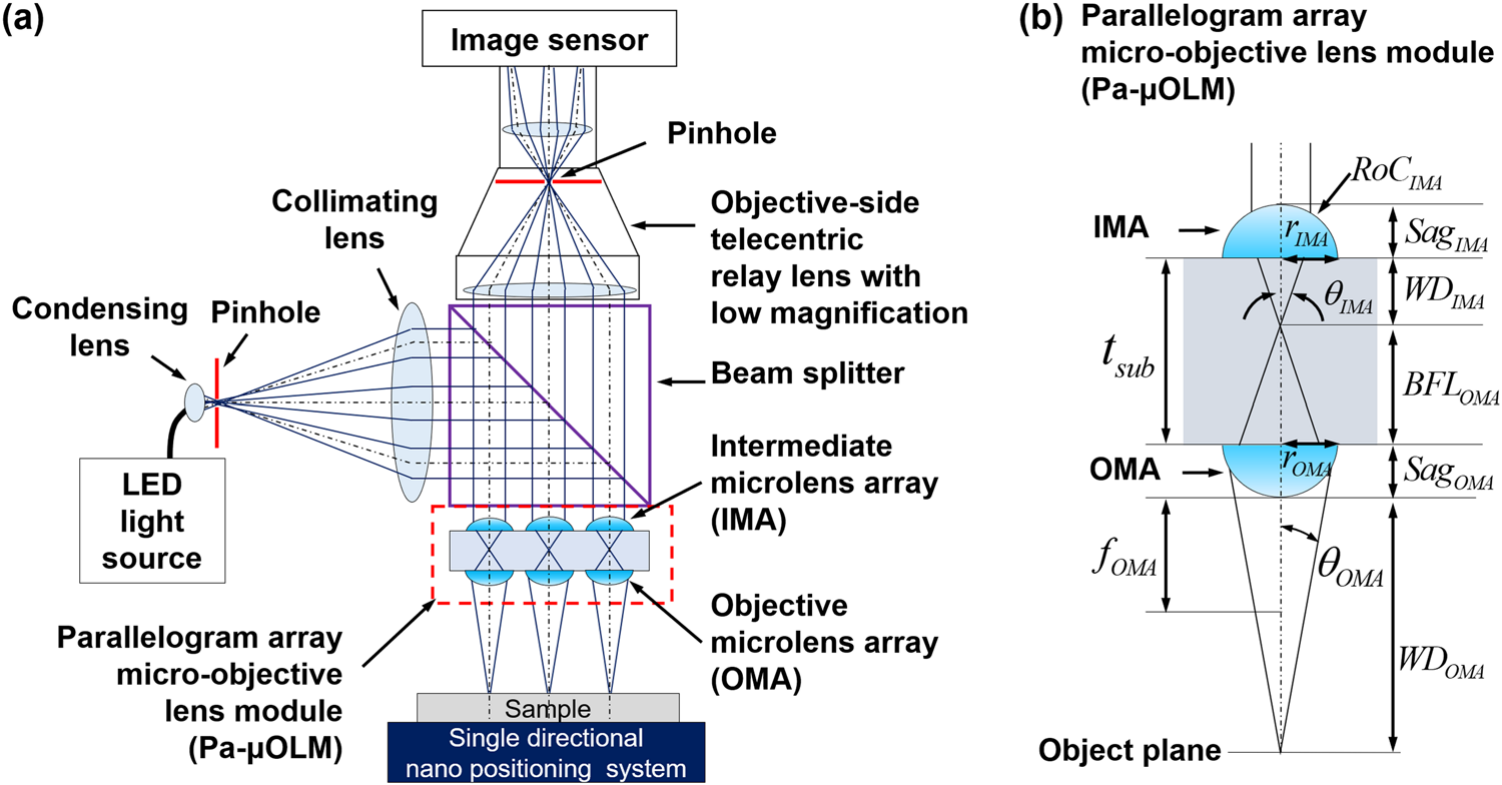
Schematic diagrams of the multi-optical probe confocal imaging system. (**a**) Schematic diagram of a gigapixel confocal imaging system using a parallelogram array micro-objective lens module (PA-μOLM) with single directional infinite scanning, and (**b**) a Pa-μOLM composed of an objective microlens array (OMA) and an intermediate microlens array (IMA).

A schematic diagram of a high-throughput confocal imaging system using multi-optical probes with single directional infinite scanning is shown in Fig. 2(a). The system consists of a Pa-μOLM composed of two parallelogram microlens arrays with a PA-MOP, beam splitter, objective-side telecentric relay lens, light source, and large-area image sensor. A point light source focuses on an objective plane after passing through the Pa-μOLM, and the optical information reflected from a sample is transmitted to the relay lens aperture located in an optically conjugated plane. This configuration allows the proposed system to operate as a confocal imaging system. The optical information reflected from the focal point on the optical axis of the OMA arrives at the image sensor after passing through the aperture of the relay lens, whereas the optical information from the OMA focal point is blocked by the aperture. Spherical aberrations introduced by the divergence of incident light are mitigated by the blocking effect imparted by the relay lens aperture.

In the proposed system, the collimated light passing through the Pa-μOLM moves through the aperture of the relay lens located in the conjugate plane. System resolution can be improved by adjusting the diameter of the aperture. The lateral resolution of the proposed system (*res*_*lateral*_) is given by

1 $$re{s}_{lateral}=\frac{0.49}{0.61}\times \frac{W{D}_{IMA}\times \,\tan ({\sin }^{-1}(\frac{{n}_{lens}}{{n}_{sub}}\times N{A}_{relay.obj}))-(Ro{C}_{IMA}-Sa{g}_{IMA})\times \,\tan ({\sin }^{-1}(N{A}_{relay.obj}))}{{m}_{OMA}}$$

Here, *WD*_*IMA*_, *Sag*_*IMA*_ and *RoC*_*IMA*_ are the WD, sag height, and radius of curvature of the IMA, respectively. *n*_*lens*_ and *n*_*sub*_ are the refractive indices of the lens and the substrate. *m*_*OMA*_ is the magnification of OMA. *NA*_*relay,obj*_ is the objective-side NA of the relay lens, as shown in Fig. 2(b). The detailed derivation of the Eq. (1) can be found in^25^.

As given by Eq. (1), *res*_*lateral*_ is improved as the aperture diameter becoming smaller, because *NA*_*relay,obj*_ fall as that diameter is reduced. However, the optical performance of the relay lens except for the aperture diameter does not affect the resolution of the system. Moreover, the uniform parallel beam generated after passing through the IMA from the object plane can only reach the center of the aperture. Thus, this configuration of the proposed system enables use of an objective-side telecentric relay lens with a low-magnification to simultaneously ensure a high lateral resolution and guarantee large-area scalability. Using a low-magnification relay lens provides additional advantages. First, by increasing the sampling size of the image sensor, the optical probe density can be increased because the number of pixels covered by each optical probe is decreased. Second, optical information acquired from each optical probe ensures adequate light intensity even if the aperture diameter of the relay lens is reduced.

To confirm the applicability of the proposed confocal imaging system with single directional infinite scanning to inspection equipment, a system including a Pa-μOLM was designed with a *i*_*transverse*_ of 1 μm/pixel and a *WD*_*OMA*_ of more than 400 μm, for inspecting patterns with a minimum line width of 2.0 μm in display circuits. This design is based on a large-area image sensor and a low-magnification objective-side telecentric relay lens.

The geometric specifications of the microlens arrays, composed of a Pa-μOLM integrated on both sides of a glass substrate with a thickness of 700 μm, were as follows. The radii and sag height of the lens arrays were 90 μm and 44.1 μm, respectively; and the focal length was 188 μm using a high-refractive index polymer with a refractive index of 1.6055 at a wavelength of 550 nm. The massively parallel array size of the Pa-μOLM with a pitch of 200 μm was 200 × 140 in the scanning and transverse directions. This configuration enables a 28.0-mm imaging scan width in the scan direction. The WD of the designed Pa-μOLM was 436.0 μm, with a *NA*_*OMA,obj*_, of 0.18. The *res*_*lateral*_ of the designed system was 1.37 μm with a *NA*_*relay,obj*_ of 0.006, as determined using Eq. (1), at an aperture diameter of 1.5 mm yielded as a confocal pinhole of 0.9 Airy disk diameter (ADD), which is adequate to block any effect of a spherical aberration generated after light passing through Pa-μOLM.

### Construction of a gigapixel confocal imaging system using a Pa-μOLM with single directional infinite scanning

**Figure 3:**
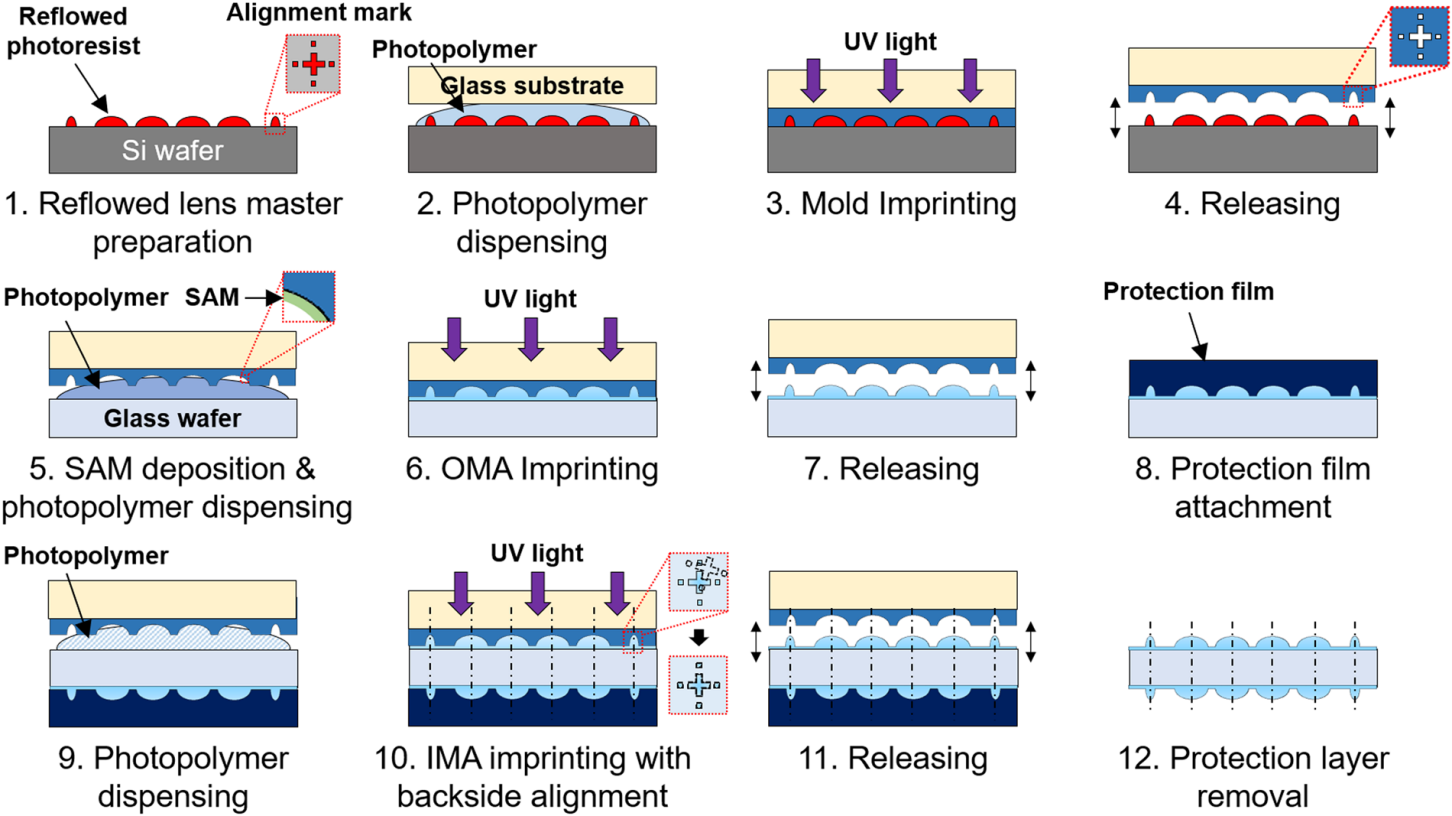
Schematic diagram of the Pa-μOLM fabrication process. The designed Pa-μOLM with two wafer-level microlens arrays was fabricated using thermal reflow and a two-step ultraviolet (UV)-imprinting process. The master microlens array and UV-transparent mold were fabricated preferentially. UV-imprinting was conducted to fabricate the OMA and IMA on both sides of a glass substrate.

Photolithography, thermal reflow and UV-imprinting processes were chosen to fabricate a wafer-level Pa-μOLM, because these processes can be guaranteed with high geometrical accuracy and mass producibility, as shown in Fig. 3. UV imprinting employing a highly durable polymer can be minimized the risks of contact and high-temperature damage to use of Pa-μOLM in an optical inspection system.

**Figure 4:**
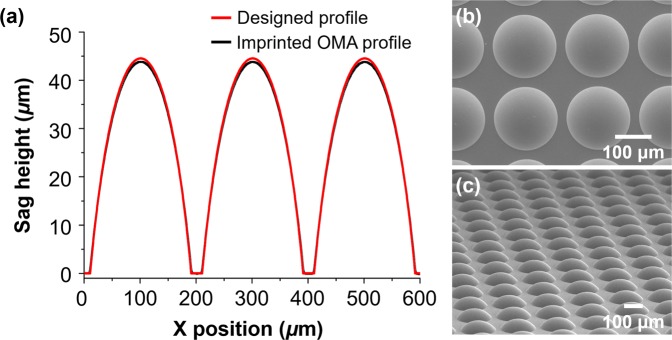
Fabrication results of the Pa-μOLM with a pitch of 200 μm, diameter of 180 μm, and sag height of 44.1 μm. (**a**) Scanning probe microscopy measurement results of the fabricated OMA on the Pa-μOLM. The geometrical deviation of the fabricated OMA from the designed value was less than 0.8%, and scanning electron microscopy images of the imprinted OMA with a pitch of 200 μm, diameter of 180 μm, and array size of 200 × 140 were obtained: (**b**) top view and (**c**) bird’s-eye view. The lens profiles were generated using OriginPro 8.0 (OriginLab Corporation, Northampton, MA, USA).

Figure 4 shows the fabrication results of the wafer-level Pa-μOLM. The OMA profiles were measured using scanning probe microscopy (Form Talysurf PGI 840; Taylor Hopson, Leicester, UK), as shown in Fig. 4(a). The geometrical deviation of the imprinted wafer-level OMA from the designed shape was less than 0.8%. Figure 4(b,c) show scanning electron microscopy images of the imprinted OMA, confirming that the microlens array was fabricated with the designed pitch, radius, and shape.

To test the compatibility of the designed system with the single directional scan method, a multi-optical probe confocal imaging system using the Pa-μOLM was constructed. A beam splitter (50-mm visible nonpolarizing cube beam splitter; Edmund Optics, Barrington, NJ, USA) was used to install the coaxial optical system between a white light-emitting device (WS-90; Haean Corp., Wonju, Korea) and an image sensor. To secure a massive number and high density of optical probes, a 71-megapixel complementary metal-oxide semiconductor (CMOS) camera (RMOD-71M; Illunis Ltd., Minnetonka, MN, USA) and an objective-side telecentric relay lens (KS_12K_TX0765; Kisoo Precision, Incheon, Korea) were integrated to achieve a sampling size of 4.05 μm/pixel for the image sensor with uniform illumination. A linear stage (SLC-24180; SmarAct GmbH, Oldenburg, Germany) was used for the single directional scan, and synchronization between the stage and camera was performed using in-house software (LabView; National Instruments, Austin, TX, USA).

### Analysis of the optical performance of gigapixel confocal imaging using a massively parallel Pa-μOLM with single directional infinite scanning

**Figure 5:**
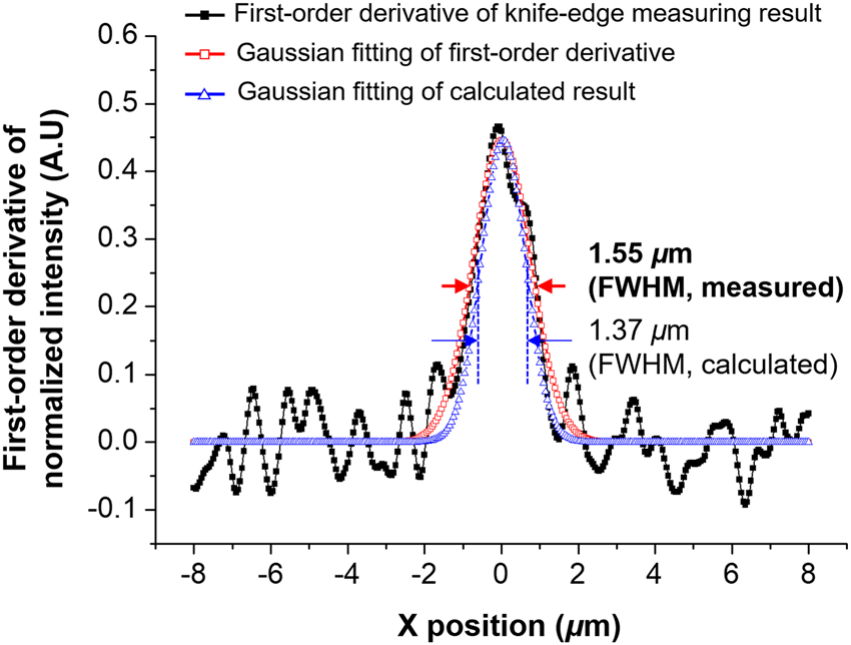
Analysis of the lateral resolution of the constructed1 optical system. Lateral resolution measured using the knife-edge detection method with the first-order derivative of the normalized intensity, and its Gaussian fitting with that of the calculated result. The lateral resolution of the proposed system was 1.55 μm, which is in good agreement with the calculated lateral resolution power of 1.37 μm. The normalized intensity profiles were generated using OriginPro 8.0 (OriginLab Corporation, Northampton, MA, USA).

A knife-edge detection method was used to quantitatively evaluate the lateral resolution of the system (*res*_*lateral*_). Images were acquired using a sampling interval of 0.02-pixel/nm. The lateral resolution was estimated by evaluating the first-order derivatives of the normalized intensity profiles and its Gaussian fitting curve, as shown in Fig. 5. The measured *res*_*lateral*_ in FWHM was 1.55 µm, with a *NA*_*relay,obj*_ of 0.006 with confocal pinhole of 0.9 ADD. Using Eq. (1), the calculated *res*_*lateral*_ of the fabricated Pa-µOLM was 1.37 μm, which shows good agreement with the measured value. To confirm that it was consistent across the imaging area, the lateral resolution was measured at several positions. The between-position deviation was less than 2.1%, indicating that the system had consistent optical performance across the entire imaging area.

**Figure 6:**
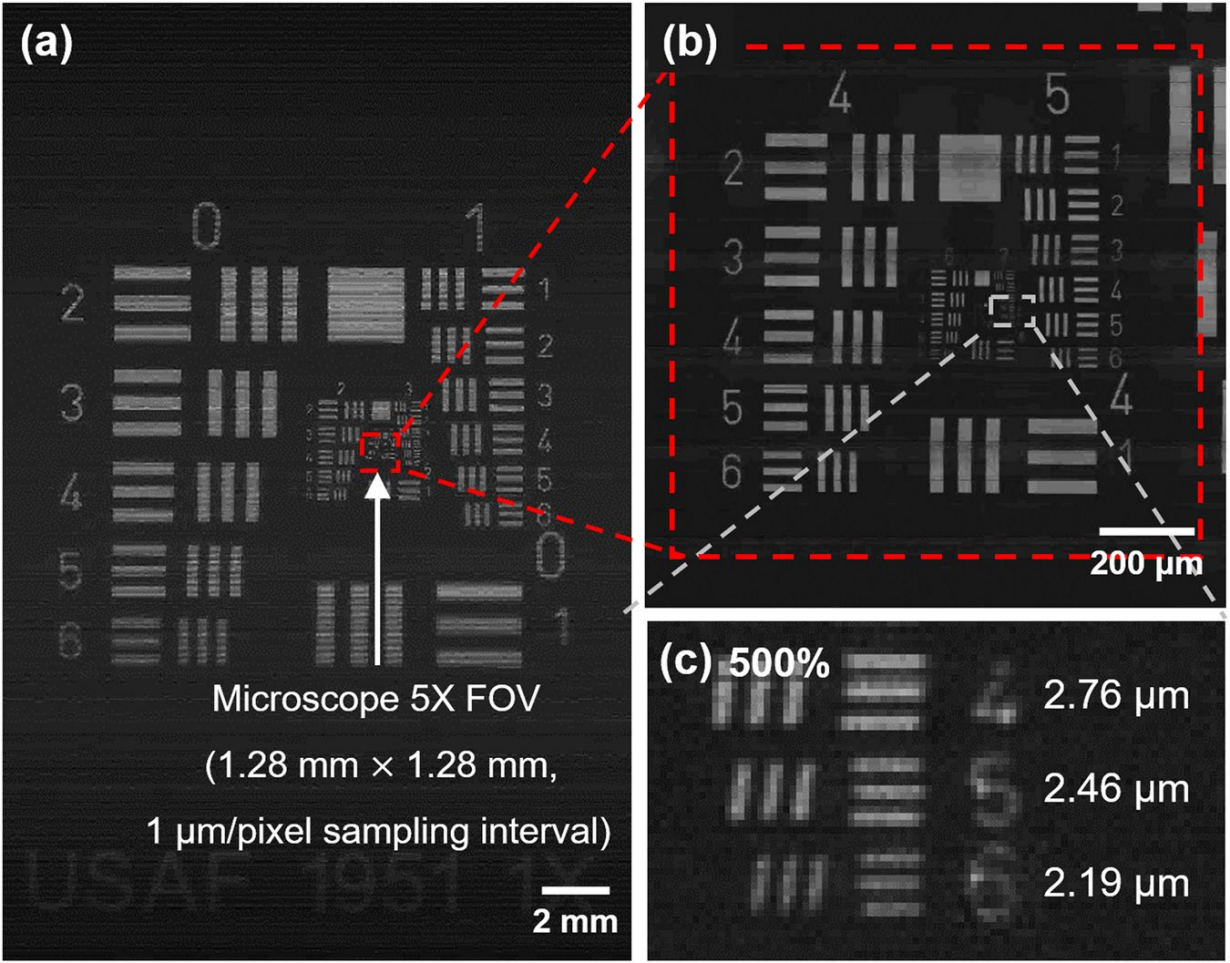
Imaging results of the USAF 1951 resolution target. (**a**) imaging results obtained using the proposed system (field-of-view (FOV): 28.0 mm × 18.5 mm; sampling interval: 1 μm/pixel), (**b**) the enlarged result in the same FOV obtained using the commercial microscope (1.28 mm × 1.28 mm), and (**c**) the enlarged result of group 7 with 500% magnification. The line for group 7 and element 6 on the test target, which has a pattern linewidth of 2.19 μm, can be easily distinguished.

The lateral resolution of the system using a single directional scan was characterized by imaging a USAF 1951 resolution target (Edmund Optics). Figure 6(a) shows the obtained target image (28.0 mm × 18.5 mm; 0.52 gigapixels). The area enclosed by the red dotted line has a size equal to the FOV of 1.28 mm × 1.28 mm under a commercial microscope (STM6; Olympus, Tokyo, Japan), with a sampling interval of 1 μm/pixel at 5× magnification. Figure 6(b) shows the enlarged result, in the same FOV, obtained by the commercial microscope shown in Fig. 6(a). Figure 6(c) shows the central region of Fig. 6(b) with 500% magnification. The line at group 7 and element 6 on the test target, which has a pattern linewidth of 2.19 μm, can be easily distinguished.

**Figure 7:**
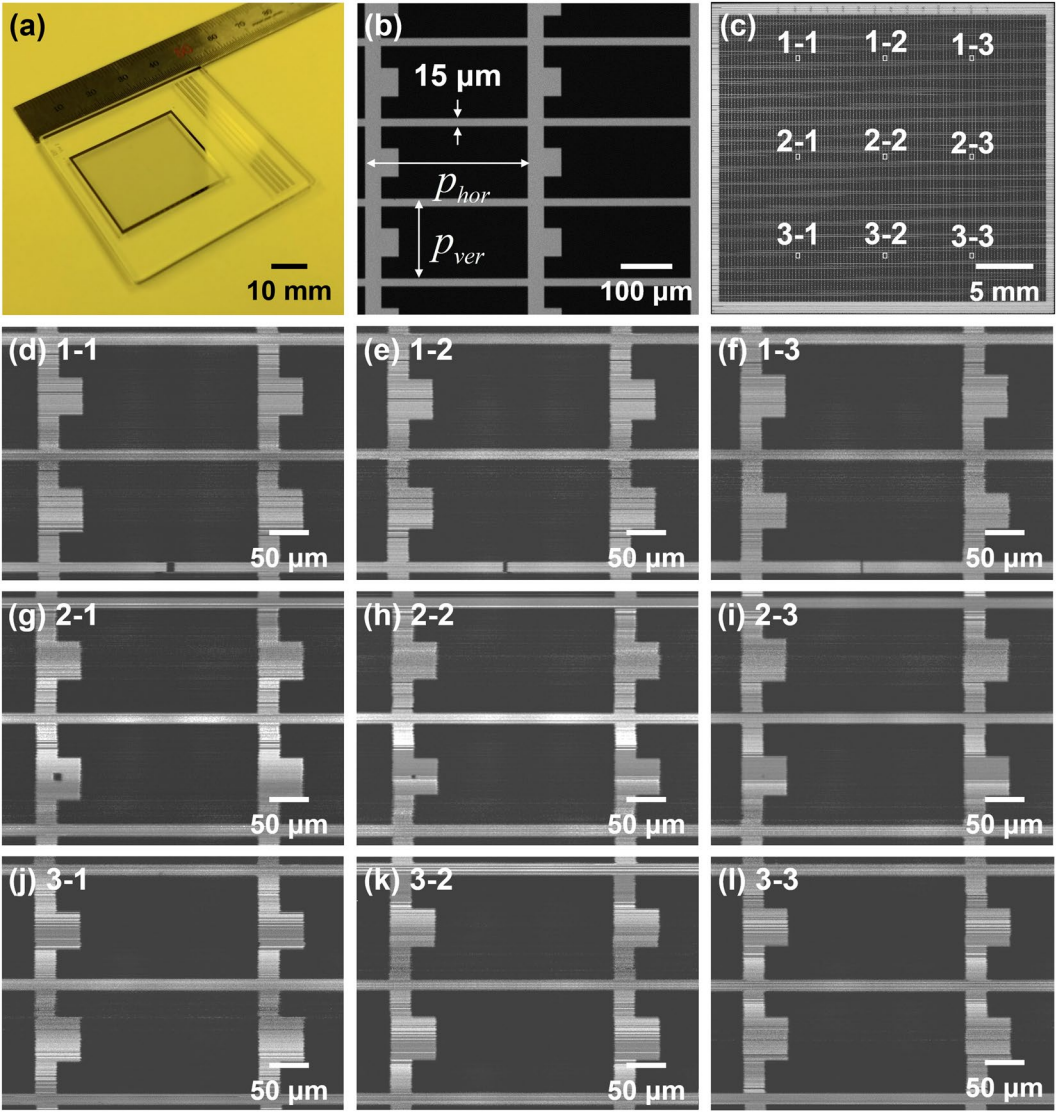
Imaging results of chrome-deposited mask for analyzing optical uniformity of the construction system. (**a**) full image of chrome-deposited photomask patterns inspired by a thin-film transistor (TFT) black matrix array in a liquid crystal display, (**b**) imaging results obtained using a conventional microscope, (**c**) mask pattern imaging result (FOV: 28.0 mm × 30.5 mm, 0.85 gigapixel image) obtained using the proposed system, and (**d**–**l**) enlarged images.

To assess the uniformity of the system in terms of its optical performance, a custom-made, chrome-deposited photomask was imaged, as shown in Fig. 7(a). A chrome-deposited photomask has patterns inspired by a TFT black matrix array on a 28.0 mm × 30.0 mm area. Figure 7(b) shows the pattern on the mask measured with the conventional microscope; the minimum linewidth of the pattern is 15.0 μm, and the horizontal pitch of the pattern (*p*_*hor*_) and vertical pitch of the pattern (*p*_*ver*_) are 280.0 μm and 140.0 μm, respectively. Figure 7(c) shows the mask-pattern imaging result (FOV: 28.0 mm × 30.5 mm; 0.85 gigapixels) obtained using the proposed system. To assess the uniformity of the optical performance, the modulation transfer function at a line 15 μm in width (*MTF*_*@15μm*_), and the pattern dimensions (*p*_*hor*_, *p*_*ver*_), were measured at nine points on a white rectangle superimposed on the imaging result. Table 1 shows the results of *p*_*hor*_, *p*_*ver*_, and *MTF*_*@15μm*_ averaged over 5 × 5 patterns at each measurement point. The *MTF*_*@15μm*_ can be calculated as follows:

2 $$MT{F}_{@15\mu m}=\frac{{I}_{\max }-{I}_{\min }}{{I}_{\max }+{I}_{\min }}$$

**Table 1:** Measured pattern dimensions and MTF at a line with a width of 15.0 μm.

Position	*p*_*hor*_ (*μ*m)	*p*_*ver*_ (*μ*m)	MTF _@ 15 μm_
1–1	280.18	140.16	0.405
1-2	279.86	140.08	0.403
1-3	279.94	139.92	0.396
2-1	280.02	140.02	0.407
2-2	280.02	140.14	0.403
2-3	280.22	139.86	0.396
3-1	279.94	140.15	0.397
3-2	280.30	140.18	0.403
3-3	280.20	139.93	0.401

Here, *I*_*max*_ and *I*_*min*_ are the brightness values of the 15.0-μm line pattern with and without the chrome pattern area, respectively. *p*_*hor*_ was 280.07 μm, with a standard deviation of 0.84 μm, while *p*_*ver*_ was 140.05 μm with a standard deviation of 1.54 μm. *MTF*_*@15μm*_ was 0.402 with a standard deviation of 0.015. These results confirmed that the constructed system with single directional infinite scanning has uniform optical performance over the entire multi-optical probe when the imaging reconstruction algorithm is used. Figure 7(d–l) shows imaging results of the white rectangular area shown in Fig. 7(c).

**Figure 8:**
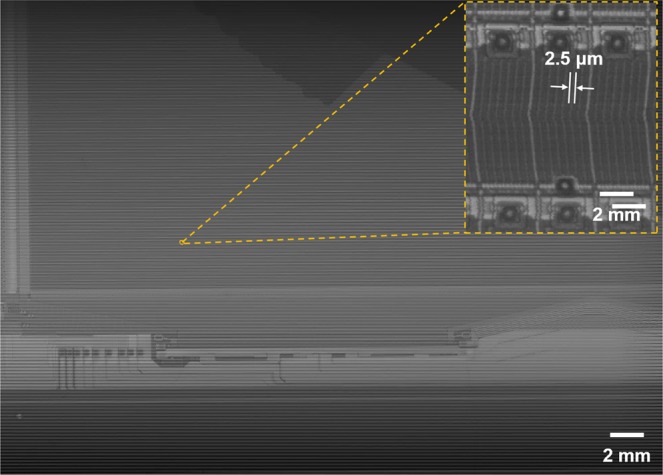
1.10 gigapixel imaging of TFT pixel glass at a resolution of 170 DPI. Imaging result of a TFT glass and a minimum linewidth of 2.5 μm. The reconstruction image was 39.7 mm × 28.0 mm in size.

To verify whether the proposed system with single directional infinite scanning can be applied to an actual display circuit, a TFT on a bottom glass for an LCD was imaged. Figure 8 shows the gigapixel imaging result of the TFT glass with a resolution of 170 dots per inch (DPI) and a minimum linewidth of 2.5 μm. The size of the reconstruction image was 39.7 mm × 28.0 mm (1.10 gigapixel image). An electronic circuit composed of various materials with different reflectance values was imaged, and the narrowest line was clearly visualized.

**Figure 9:**
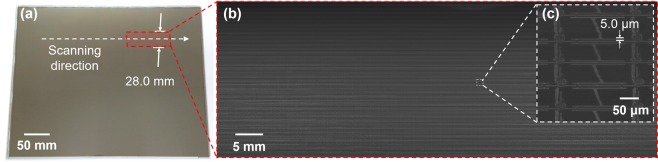
2.23 gigapixel imaging result of TFT glass at a resolution of 100 DPI. (**a**) Sample image at 392 mm × 300 mm; (**b**) the imaging result of LCD TFT glass. The reconstruction image was 79.5 mm × 28.0 mm, and (**c**) enlargement of a small area of (**b**). The minimum linewidth of 5.0 μm was clearly visible over the entire imaging area.

Finally, scalability of the single directional infinite scanning in terms of in-line optical inspection was confirmed via imaging of a large-area TFT-bottom glass. Figure 9 shows the imaging results for a large area TFT-bottom glass with a resolution of 100 DPI. The sample size was 392 mm × 300 mm; TFTFs were patterned over the entire area, as shown in Fig. 9(a). Figure 9(b,c) show the imaging results as obtained and at 500% magnification. The reconstruction image was 79.5 mm × 28.0 mm in dimensions (2.23 gigapixel image), and the minimum linewidth of 5.0 μm was clearly visible over the entire area. The system works in practice, affording large-area scalability and high-lateral resolution.

Therefore, the high-throughput gigapixel confocal imaging system using massively parallel multi-optical probes with a single directional scan can be applied for inspection of electronic circuits.

## Discussion

We propose a gigapixel confocal imaging system, with a single directional scan using a massively parallel Pa-μOLM, to allow simultaneous high-lateral resolution and large-area inspection. A Pa-MOP was applied to a single directional scan strategy for improvement of imaging throughput based on a multi-optical probe. A Pa-μOLM was designed and integrated into a confocal imaging system using an objective-side telecentric relay lens with a low-magnification, to guarantee high lateral resolution and increase the WD. A massively parallel Pa-μOLM was fabricated through a UV-imprinting process to secure high-durability mass producibility and high geometrical accuracy at the wafer-level. After construction of the confocal imaging system, the knife-edge detection method was used for quantifying the lateral resolution, to evaluate the optical performance of the proposed system. Imaging tests were also performed to verify the feasibility of the proposed scan strategy.

The proposed system overcomes the limitations related to imaging throughput for large-area, high-lateral resolution measurements, by incorporating a single directional scan using a Pa-μOLM and a low-magnification relay optical system. Notably, a low-magnification, objective-side telecentric lens is used to ensure adequate imaging brightness; we employ high-density massively parallel optical probes to expand the imaging area. The unit cost of optical system integration is reduced, and the imaging area can be easily extended without reducing throughput.

The Pa-μOLM consisted of an IMA and OMA with a pitch of 200 μm, diameter of 180 μm, and sag height of 43.8 μm, arranged in a parallelogram-shaped array on both sides of a glass substrate. A spherical microlens was fabricated with a geometrical deviation of less than 0.8% relative to the designed lens, and exhibited a WD of 445 μm. The FWHM of the lateral resolution measured by the knife-edge detection method was 1.55 μm. Tests using the USAF 1951 resolution target and the chrome-deposited mask were performed for a single directional scan with a gigapixel imaging with a width of 28.0 mm, to evaluate the optical performance of the system. System feasibility and scalability in terms of optical inspection were confirmed via gigapixel imaging of a real-world, large-area TFT bottom glass for an LCD. The results confirmed that the proposed system with single directional infinite scanning could be used to inspect actual large-area electronic circuits with high throughput.

In the present study, the imaging speed was limited, as the number of sensor pixels far exceeded the number of optical probes. The inspection speed could be improved dramatically by integrating the proposed system with a dedicated sensor capable of 1:1 matching with the optical probe.

## Methods

### Fabrication of the wafer-level Pa-μOLM

A positive photoresist (AZ9260; Microchemicals GmbH, Ulm, Germany) was coated on a 6-in silicon substrate (LG Siltron, Inc., Seoul, Korea) with a thickness of 26.2 μm, and prebaked on a hotplate for 240 s at 110°C. Micro-cylindrical pedestal arrays with a radius of 90 μm were fabricated via photolithography using a quartz mask and a mask aligner (MA-6; SUSS MicroTec GmbH, Garching, Germany). The arrays were exposed to I-line UV light at a dose of 1,700 mJ/cm^2^, and then immersed in a developer (AZ-400k; Microchemicals GmbH) for 300 s. A positive master of the spherical microlens array with a sag height of 43.8 μm was obtained via thermal reflow for 15 s at 160 °C using the prepared micro-cylindrical pedestal array.

A negative transparent mold was preferentially prepared via a UV-imprinting process for fabrication of the final Pa-μOLM with a high-refractive index polymer. The mold was transparent for the optical backside alignment process, to allow alignment of the center axis of the lenses on both sides of the glass substrate. A UV-curable photopolymer (MINS-ERM; Changsung Sheet, Paju, Korea) was coated on the master and polymerized using broadband UV light exposure, at a dose of 3,500 mJ/cm^2^, after covering a 6-in transparent substrate. After releasing the mold from the master, a self-assembled monolayer of Trichloro (1 H, 1 H, 2 H, 2H-perfluorooctyl) silane (Sigma-Aldrich, St. Louis, MO, USA) was coated on the negative mold for enhancement of the releasing property on the surface. A UV-imprinting process using a high refractive index UV-curable photopolymer (Ormoclear HI01XP; Microresist Technology GmbH, Berlin, Germany) was conducted to fabricate Pa-μOLM on a glass substrate with thickness of 700 μm. The photopolymer was coated onto the glass by applying the mold and polymerized via 3,000 mJ/cm^2^ broadband UV light exposure. The replicated microlens array was then released from the mold, and a film for protection of the replicated lenses was attached. The microlens array on the opposite side was replicated using the same UV-imprinting process. The center axis of the microlens on each side was aligned via an optical backside alignment process. Finally, the protective film was removed.
